# Draft genome sequences of *Listeria monocytogenes* strains of sequence type 1733 which constituted a pseudo-outbreak due to contaminated blood agar media

**DOI:** 10.1128/mra.00732-23

**Published:** 2023-12-15

**Authors:** Sangmi Lee, Asmaa Sadat, Zuzana Kucerova, Sophia Kathariou

**Affiliations:** 1 Department of Food and Nutrition, Chungbuk National University, Cheongju, Chungbuk, South Korea; 2 Department of Bacteriology, Mycology, and Immunology, Mansoura University, Mansoura, Dakahlia, Egypt; 3 Division of Foodborne, Waterborne, and Environmental Diseases, Centers for Disease Control and Prevention, Atlanta, Georgia, USA; 4 Department of Food, Bioprocessing, and Nutrition Sciences, North Carolina State University, Raleigh, North Carolina, USA; Indiana University, Bloomington, Bloomington, Indiana, USA

**Keywords:** *Listeria monocytogenes*, pseudo-outbreak, ST1733, lineage III

## Abstract

We report genome sequences of *Listeria monocytogenes* sequence type (ST) 1733 from a 2013 pseudo-outbreak, where *L. monocytogenes* isolation from non-sterile sites (urine, wound, or abscess) was an artifact from contaminated sheep blood in the isolation media. Two ST1733 strains from wound and urine in 2005 are also reported.

## ANNOUNCEMENT

*Listeria monocytogenes* is the foodborne etiological agent of severe disease (listeriosis) in humans (1 – 3), which is typically confirmed by isolation of this bacterium from normally sterile sites such as blood (4). Strains from urine are uncommon (5). *L. monocytogenes* from clinical specimens is typically isolated on agar media containing sheep blood (4). If the blood is contaminated with *L. monocytogenes*, e.g., when derived from animals with listeriosis, isolation of *L. monocytogenes* can be an artifact of the media, leading to pseudo-outbreaks (4). In 2013 and 2014, *L. monocytogenes* strains from non-sterile sites were found to constitute such pseudo-outbreaks (4). We report the draft genomes of three strains isolated in 2013, all of lineage III, sequence type (ST) 1733 that were initially presumed to originate from urine, wound, and abscess samples but actually resulted from contamination of sheep blood media (Table 1). Interestingly, two earlier ST1733 strains were isolated during analysis of wound and urine samples in 2005, and their draft genomes are also reported (Table 1).

**TABLE 1:** Characteristics of the genomes from the *L. monocytogenes* ST1733 strains examined in this study

Characteristics	PNUSAL002365^*a*^ ^,^ ^*b*^	PNUSAL002366^*a*^ ^,*b*^	PNUSAL000827^*a*^ ^,^ ^*c*^	J3412^*d*^	J3498^*d*^
Isolation year, country (state)	2013, USA (CA)	2013, USA (CA)	2013, USA (MI)	2005, USA (NV)	2005, USA (MI)
Source	Human (urine)	Human (wound)	Human (abscess)	Human (abscess wound)	Human (urine)
GenBank accession no.	AAATYV000000000.1	AAATYP000000000.1	AAAIAN000000000.1	AAAOVJ000000000.1	AAAZCH000000000.1
BioProject accession no.	PRJNA212117	PRJNA212117	PRJNA212117	PRJNA212117	PRJNA212117
BioSample accession no.	SAMN05755934	SAMN05755935	SAMN02924346	SAMN03760836	SAMN04536625
Assembly accession no.	PDT000157442.2	PDT000157441.2	PDT000035533.3	PDT000066113.2	PDT000112613.2
Raw reads accession no.	SRR5000713	SRR5000712	SRR1520078	SRR2049101	SRR3210347
Sequencing method	MiSeq	MiSeq	MiSeq	MiSeq	MiSeq
Number of reads (pairs)	1,275,664	1,189,991	1,011,106	1,023,630	1,097,122
Total length of reads (Mbp)	361.7	333.3	300.1	301.2	323.8
Draft genome length (Mbp)	3.1	3.1	3.1	3.0	3.1
Average coverage (×)	117	107	96	99	105
Average GC content (%)	37.5	37.5	37.5	37.5	37.5
Number of contigs	107	63	23	43	20
N50 (bp)	90,082	216,497	374,455	230,829	511,456
Protein coding sequences	3,057	3,064	3,078	2,965	3,003
Pseudogenes	52	53	54	48	49
rRNAs	7	6	7	7	6
tRNAs	57	50	53	52	54

^*a*^ Strains were from the 2013 pseudo-outbreak reported by Matanock et al. (4).

^*b*^ No cgMLST allele differences identified; 38–49 cgMLST allele differences from the other strains.

^*c*^ cgMLST analysis revealed 24–49 allele differences between PNUSAL000827 and the other strains.

^*d*^ Two cgMLST allele differences. Pairwise comparisons with the other strains showed 24–40 cgMLST allele differences.

Strains were isolated by state health departments in the United States on sheep blood agar and identified using traditional methods with biochemical tests. Suspected *L. monocytogenes* isolates were shipped to the Centers for Disease Control and Prevention (CDC) and confirmed via the AccuProbe *L. monocytogenes* culture identification test (Hologic, Marlborough, MA, USA). Genomic DNA was extracted with the DNeasy blood and tissue kit (QIAGEN, Valencia, CA, USA) from bacteria grown overnight at 37°C on BD BBL Trypticase Soy Agar with 5% Sheep Blood (Becton, Dickinson and Company, Kelberg, Germany). Paired-end libraries were constructed using 0.5–1 ng of genomic DNA with the Nextera XT DNA library preparation kit (Illumina, San Diego, CA, USA) and sequenced with Illumina MiSeq (2 × 150 bp; Table 1). BIONUMERICS (version 7.6.3, Applied Maths NV, Saint-Martens-Latem, Belgium) was utilized to check read quality and overrepresented sequences, trim adaptors, and filter low-quality reads (average quality ≥30, average *de novo* coverage ≥20×, genome length between 2.8 and 3.1 Mbp, and percent core present ≥95%). The reads were assembled with SKESA (version 2.2) (6), and the contigs were annotated via the NCBI Prokaryotic Genome Annotation Pipeline (version 4.5 for strains J3412 and PNUSAL000827 and version 4.7 for the other strains) (7) (Table 1). ST designations were obtained from the draft genomes using BIGSdb-Lm maintained by Institut Pasteur, and genomes were compared with cgMLST using Genome Comparator (https://bigsdb.pasteur.fr/cgi-bin/bigsdb/bigsdb.pl?db=pubmlst_listeria_isolates&page=plugin&name=GenomeComparator) (8). Default parameters were employed for all the analysis tools unless otherwise specified. Lineage III strains have a proclivity for non-human animals, especially ruminants (9), and we hypothesize that the sheep from which the blood originated had been infected by *L. monocytogenes* ST1733. The ST1733 strains from 2005 and 2013 were closely related, with ≤49 cgMLST differences (Table 1), suggesting that the strains from 2005 also resulted from contaminated media and long-term presence of the ST1733 strains in the sheep production system that supplied the blood.

## Data Availability

The genome sequences were deposited at GenBank under the accession numbers shown in Table 1.
